# [Retracted] Predictors of efficacy of anamorelin in patients with non‑small cell lung cancer and cachexia: A retrospective study

**DOI:** 10.3892/ol.2024.14360

**Published:** 2024-03-26

**Authors:** Yoshiko Ishioka, Hisashi Tanaka, Tomonori Makiguchi, Syunsuke Fujishima, Yasuhito Nunomura, Hiroaki Sakamoto, Toshihiro Shiratori, Kageaki Taima, Sadatomo Tasaka

Oncol Lett 27: 22, 2024; DOI: 10.3892/ol.2023.14154

Following the publication of the above paper, the authors have submitted a request to the Editorial Office to retract the article on account of a problem regarding ethical approval. The analysis was performed on patients who received anamorelin between July 2021 and November 2022; however, upon re-examining the ethical approval statement, it was stated to be applicable only for patients who received anamorelin between July 2021 and April 2022. Owing to this oversight on the part of the authors, patients were also included in the study over the period April-November 2022, which was not covered by the ethical approval.

Given that this problem has come to light, the Editor of *Oncology Letters* has agreed to the authors’ request to retract this paper from the Journal. The authors and the Editor apologize to the readership for any inconvenience caused.

